# What’s wrong with John? a randomised controlled trial of Mental Health First Aid (MHFA) training with nursing students

**DOI:** 10.1186/s12888-017-1278-2

**Published:** 2017-03-23

**Authors:** Sharyn Burns, Gemma Crawford, Jonathan Hallett, Kristen Hunt, Hui Jun Chih, P.J. Matt Tilley

**Affiliations:** 1 Collaboration for Evidence, Research and Impact in Public Health, School of Public Health, Faculty of Health Sciences, Curtin University, GPO Box U1987, Perth, WA 6845 Australia; 2 School of Public Health, Faculty of Health Sciences, Curtin University, Perth, Australia

**Keywords:** University, Nursing students, Australia, Mental health literacy, Prevention and early intervention, Mental Health First Aid, Recognition of depression, Stigmatising attitudes, Social distance, Depression

## Abstract

**Background:**

The prevalence of mental health problems have been found to be higher among university students compared to their non-student peers. Nursing students in particular face a range of additional stressors which may impact their undergraduate performance and their careers. Mental Health First Aid (MHFA) aims to increase mental health literacy and to reduce stigma and may positively impact on the student population. This paper describes a MHFA randomised controlled trial targeting nursing students at a large Australian university. This study aimed to measure the impact of the MHFA course on mental health literacy, mental health first aid intentions, confidence in helping someone with a mental health problem and stigmatising attitudes including social distance.

**Methods:**

Participants were first year nursing students (*n* = 181) randomly allocated to the intervention (*n* = 92) or control (*n* = 89) group. Intervention group participants received the standardised MHFA course for nursing students. Online self-report questionnaires were completed at three time intervals: baseline (one week prior to the intervention: T_1_) (*n* = 140), post intervention (T_2_) (*n* = 120), and two months post intervention (T_3_) (*n* = 109). Measures included demographics, mental health knowledge, recognition of depression, confidence in helping, mental health first aid intentions and stigmatising attitudes including social distance. Repeated measures ANOVA was computed to measure if the impact of time (T_1_, T_2_, T_3_) and group (intervention and control) on the outcome variables.

**Results:**

There was a significant improvement among intervention compared to control group participants across the three time periods for knowledge scores (*p* < 0.001), confidence in helping (*p* < 0.001), mental health first aid intentions (*p* < 0.001), total personal stigma (*p* < 0.05), personal dangerous/unpredictable stigma (*p* < 0.05) and social distance (*p* < 0.05) scores.

**Conclusion:**

MHFA is useful training to embed in university courses and has the potential to enhance mental health literacy and reduce stigmatising attitudes and social distance. While this course has particular salience for nursing and other health science students, there are broader benefits to the general university population that should be considered and opportunities accordingly explored for all students to complete the course.

**Trial registration:**

Australian New Zealand Clinical Trials Registry ACTRN12614000861651. Retrospectively registered 11 August 2014.

## Background

The burden of poor mental health in Australia is substantial, with almost half of Australians developing a mental illness at some point in their life [1]. Poor mental health can have long term impact across the lifespan, affecting educational achievement, social interactions, employment and contribution to community [1, 2]. In addition, poor mental health is associated with substance misuse, increased risk of violence and poor reproductive and sexual health [1, 2]. Mental health problems are most likely to manifest before the age of 25 years [1] representing the age period for the majority of undergraduate university students [3].

### Mental health in university students

Australian university students report higher levels of psychological distress than their non-student peers [4], and face unique mental health issues [5–7]. A systematic review (*n* = 24 studies) found a weighted mean prevalence of depression of 30.6% among undergraduate university students [8]. Financial strain and lower socio economic status, being in first or second year of study, being female, and living off campus or alone increases the risk of poorer mental health outcomes for students [4, 5, 7, 9, 10]. Poor mental health can have a significant impact on educational and academic performance, student retention, and future employment [11–15] with one study for example finding a significant decrease in academic performance among students with diagnosed depression [16].

Uptake of mental health services among university students has been found to be poor, with one Australian study finding up to 84% of students that met criteria for depression (Patient Health Questionnaire-9) and anxiety (Patient Health Questionnaire anxiety module) did not access services [17]. Despite most students in the study having health insurance and access to a range of free services available on campus, reasons for lack of help seeking included scepticism about treatment, a perceived lack of need and lack of awareness of health care availability and insurance cover [17]. It is acknowledged that young people are less likely to seek help for mental health problems if they have negative attitudes towards seeking help, if they feel they should be able to resolve the issue themselves and if they are experiencing suicidal ideation [18]. Accessing help for mental health problems is recognised as a protective factor [18] with indicated and early intervention programs likely to be most beneficial to adolescents and young adults [18, 19].

### Mental health and nursing students

Although nursing students face similar stressors to other tertiary students, additional factors have been identified which place them at risk for developing mental health problems [20]. Stress relating to academic and time pressures, high workloads, work placements and the responsibility and experience of patient care [20, 21] not only affect nursing students while at university but further, mental health issues experienced at an undergraduate level may negatively impact future careers [21]. Within the workplace nurses require continuous interaction with patients, family members and other health professionals in high stress environments [22]. Prolonged exposure to this type of work environment can negatively affect mental health which in turn may lead to absenteeism, high turnover of staff and burnout [23].

Mental health programs targeting nursing students have been found to positively influence their attitudes towards mental health, increase social supports and health literacy and decrease stigma [21, 24]. Utilising social supports has had a range of positive outcomes on the mental health of students including lowering stress; increasing coping mechanisms for stress; and promoting positive wellbeing [21]. An Australian study found structured programs examining mental health issues increased nursing students’ confidence and preparedness to respond to mental health issues [25]. Further, the program was found to better prepare students for the workplace and provided nurses with the ability to recognise the early signs and symptoms of mental illness amongst their colleagues and peers [23].

### Building mental health literacy

There is a recognised need to enhance mental health literacy among the university population [6] and among allied health [26] and nursing students [24]. Mental health literacy has been identified as an important component of a nursing degree and it is recommended that it is introduced early in the course [24]. Targeting nursing students during their first year of university provides the opportunity to enhance mental health literacy and decrease stigma among nursing students, to provide a basis for further mental health courses within their degree and to enhance their support of peers who may be experiencing a mental health issue [27, 28].

Mental Health First Aid (MHFA) is an internationally recognised training course which provides participants with the skills to identify and support those who may be developing a mental health problem or in a mental health crisis [29]. The course has been identified as a potential intervention for health professionals including nursing, medical [27] and pharmacy students [26] and for other support services [30] to improve mental health literacy and increase the likelihood of providing mental health first aid to a peer experiencing a mental health problem. In 2013 the MHFA Australia’s Standard MHFA course was tailored to specifically target the needs of nursing and medical students, with the addition of an eating disorders component found in the Youth MHFA course (for adults working or living with adolescents) [27].

This paper describes the results of a pragmatic, waitlisted randomised controlled trial (RCT) to measure the impact of MHFA training with nursing students at a large university in Perth, Western Australia. This study adds to the growing literature regarding MHFA specifically for program targeting health professionals such as nursing students. Impact was assessed by mental health literacy measures including recognition of depression and knowledge of mental health issues [29], mental health first aid intentions, confidence in helping someone with a mental health problem and stigmatising attitudes including social distance. We hypothosized that participants in the intervention group would report higher levels of mental health literacy and mental health first aid intentions, greater confidence in helping and lower stigmatising attitudes scores compared to control group participants.

## Methods

### Participants

Eligible participants were first year undergraduate nursing students completing a practical unit (approximately *n* = 250); enrolled in the internal study mode; studying at the main campus; and aged 18 years or older. The university student intranet, an in-class presentation delivered by one of the research team members, and fliers were used to advertise the study. Participants were provided the MHFA course at no charge (control group participants were able to complete the online course after the T_3_ data collection period) and were able to use course participant for a portion of volunteer hours required for the practical unit. No other incentives were provided.

**Fig. 1:**
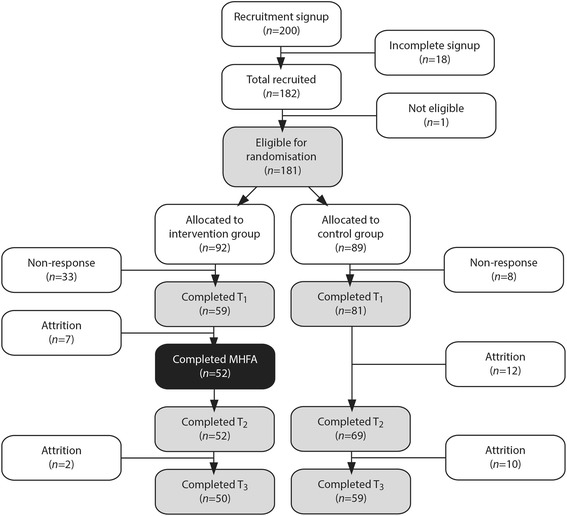
Trial schema

As the literature [29, 31] suggested a high correlation of 0.8 for the baseline-post measurements, it was necessary to have 50 students in each the intervention and the control group to detect 5% level of significance with 90% power in order to detect medium effect sizes in the outcome variables [28]. For the purpose of the power analysis mental health knowledge was selected as the key outcome variable. Based on the findings of Jorm et al. [29] a conservative 1.5 difference between baseline and post intervention knowledge scores was estimated. Participation in university studies has been found to be low [32, 33]. In order to ensure an adequate sample size for both the intervention and control groups at the completion of the study all undergraduate students enrolled in the designated unit (approximately *n* = 250) were invited to register interest. Students were advised of potential dates for the MHFA course and of the eligibility criteria hence reducing the likelihood of recruiting ineligible participants. A total of 200 students initially expressed interest in taking part in the study. The recruitment was influenced by the practicalities of working within a large university which required delivering the intervention during semester at a time convenient to university scheduling and clinical rotations for students (see Fig. 1).

### Procedure

After online registration of interest (*n* = 200) students who successfully completed registration and who were eligible (*n* = 181) were randomly assigned to either the intervention or control group using computer generated automated randomisation and were notified of their intervention condition (intervention *n* = 92; control *n* = 89). The computer generated randomisation and notification was implemented by a Research Assistant Consent was obtained from all participants at the beginning of the baseline questionnaire (intervention *n* = 59; control *n* = 81). Due to logistics involved in attending the face-to-face course and the need to provide volunteer hours for participation in the intervention in a specific unit during the university semester, participants were aware of allocation to intervention or control group at the time of completion of the baseline questionnaire. Consent was obtained from all participants at the beginning of the baseline questionnaire. More detail regarding the research methods can be found in the study protocol [28]. This study follows the CONSORT Guidelines [34] for the design and implementation of randomised controlled trials. This study was approved by the Curtin University Human Research Ethics Committee (SPH-74-2013).

#### The intervention

Intervention group participants received the tailored MHFA course for nursing students [28]. The training included: signs, symptoms and risk factors for common mental health problems, including depression, anxiety, substance use disorders, psychosis and eating disorders and strategies to assist someone experiencing a number of mental health crises [28]. Two face to face courses were delivered during semester by accredited MHFA facilitators. Each course comprised two-6.5 h sessions run over two days. All participants were provided the MHFA manual [35], standardised MHFA materials including specific materials designed for nurses. MHFA is a standardised course [36, 37], facilitated by accredited professionals therefore enhancing the fidelity of the course and reducing risk of Type 111 error [38]. The control group received no intervention during the data collection period (baseline: T_1_ to two months post intervention: T_3_). Once control group participants completed the T_3_ questionnaire they were offered the online version of the MHFA course. The online course was completed by 64 study participants within one month of T_3_ data collection.

Participants completed an online self-report questionnaire at three time intervals: baseline (one week prior to the intervention): (T_1_), post intervention (T_2_), and two months post intervention follow up (T_3_). Demographics questions were excluded in the post intervention and follow up questionnaires. Two months post intervention was selected for follow up data collection to work within the constraints of university semesters and clinical rotations. Four reminder emails were sent to non-completers over a two week period (see Fig. 1).

### Measures

Data were collected to measure demographics, mental health knowledge, recognition of depression, confidence in helping, mental health first aid intentions and stigmatising attitudes including social distance.

#### Mental health knowledge, recognition of depression and confidence

Mental health knowledge was assessed using 20 statements for which students were asked to respond ‘true’ or ‘false’. These questions were adapted from previously validated MHFA knowledge statements [29]. A score (0–20) was computed with a higher score indicating better knowledge**.**

The questionnaire used the vignette described below to guide questions associated with recognition of mental health problems, confidence assisting with mental health, social distance, and personal and perceived stigma. The vignette was adapted from previous questionnaires [26, 29] and meets the diagnostic criteria set by the DSM-IV [39] and ICD-10 for major depression [40] and is described below:

*John is a 21-year-old student who has been feeling unusually sad and miserable for the last few weeks. He is tired all the time and has trouble sleeping at night. John doesn’t feel like eating and has lost weight. He can’t keep his mind on his studies and his marks have dropped. He puts off making any decisions and even day-to-day tasks seem too much for him. His parents and friends are very concerned about him. John feels he will never be happy again and believes his family would be better off without him. John has been so desperate, he has been thinking of ways to end his life.*

Similar to other studies [27] to measure recognition of depression participants were provided the vignette then asked an open-ended question “*what, if anything is wrong with John?”* Responses of ‘depressed’, ‘depression’, ‘affective disorder’ or ‘mood disorder’ signified a correct identification of John’s problem.

#### Mental health first aid intentions

Mental health first aid intentions was measured by an open-ended question which asked participants to: *Imagine John is someone you have known for a long time and care about. You want to help him. What would you do?* This was scored by a research assistant then ratified by three researchers using the scoring system described by Yap and Jorm [41]. This system is based on the ALGEE action plan which is a key focus of the MHFA course [35]. Responses may receive 0–2 points per component of the ALGEE (*Approach the person; Assess and Assist with any crisis; Listen non-judgementally; Give support and information; Encourage appropriate professional help; and encourage other supports*) (Total score 0–12) [42].

To assess confidence to assist someone with a mental health problem, participants were asked about their confidence in their ability to help John. Responses included a five point Likert scale ranging from: 1 (don’t know) to 5 (very confident) (Score 0–5).

#### Stigmatising attitudes

MHFA and other community-based studies have consistently used the Depression Stigma Scale (DSS) [43] and the Social Distance Scale (SDS) [44] to measure the constructs of stigmatising attitudes. While the DSS measures beliefs about people with mental health illnesses, the SDS measures intended avoidance behaviour [45]. As for other studies [27, 45, 46], due to the complexity of measuring these constructs this study measured personal and perceived stigma and social distance.

Personal and perceived stigma were measured using a seven item scale which was adapted from the validated Depression Stigma Scale (DSS) [43]. Respondents were asked two sets of questions, the first about their personal feelings towards John and the second about their perception of others’ feelings towards John. Consistent with other MHFA studies [46] some questions were excluded, for example, voting for John as a politician, and the wording was modified. Personal stigma statements included: ‘*John could make himself better if he wanted,*’ ‘*John’s problem is a sign of personal weakness*,’ ‘*John’s problem is not a real medical illness*,’ ‘*John is dangerous*,’ ‘*It is best to avoid John so that you don’t develop this problem yourself*,’ ‘*John’s problem makes him unpredictable*,’ and ‘*You would not tell anyone if you had a problem like John’s’*. Responses were scored on a five point Likert scale from “strongly agree” to “strongly disagree”. The perceived stigma questions used the same questions, response items and scoring method however participants were asked to consider ‘what you think most other people believe’. Personal and perceived stigma scores were computed and divided by seven with higher scores representing lower stigmatising attitudes.

Social distance was measured by a scale adapted from the previously validated Social Distance Scale (SDS) [44] and used in other MHFA studies [27, 46]. Participants were asked to respond to five statements asking how they would feel spending time with John. A five point Likert scale was used with responses ranging from “definitely not” through to “definitely”. Students were asked if they would be happy to ‘*go out with John on the weekend*’, ‘*to work on a project with John*,’ ‘*to invite John around to your house*,’ ‘*to go to John’s house*,’ and to ‘*develop a close friendship with John’*. Scores were computed then divided by 5 to derive a mean [44] with higher scores representing less social distance and lower stigmatising attitudes [47].

### Data analysis

A Principal Component Analysis with varimax rotation was employed to reduce stigma and social distance items. Previous studies have employed Principal Component Analysis [29] and Exploratory Structural Equational Modelling [45] to reduce stigma and social distance items. While components are similar across studies there is some variation. For example, the study by Jorm and Wright [46] included a ‘social distance’, ‘stigma perceived in others’, ‘dangerous and unpredictable’ and a ‘weak-not sick’ component. A fifth component ‘reluctance to disclose’ which included the single item *‘you would not tell anyone if you had a problem like John’s’* was also included. A study by Yap et al. [45] also include a social distance component, in addition to ‘personal weak not sick’ ‘personal dangerous/unpredictable’, perceived weak not sick’ and perceived weak not sick’. Measuring personal and perceived stigma separately was seen to be important for community interventions [45]. Yap et al. [45] found the ‘reluctance to disclose’ item to load moderately on to the personal and the perceived ‘dangerous/unpredictable’ factors for the general community, but not for the youth survey. The personal and perceived items about ‘it is best to avoid people like John so you don’t develop this problem yourself’ to load only moderately onto both the ‘weak not sick’ and the ‘dangerous/unpredictable’ factors [45]. Others have also found weak but moderate loadings for this variable onto both of these factors [48]. This study found a weak moderate loading for the perceived but not for the personal stigma factors. In the initial analysis the addition of *‘it is best to avoid people like John so you don’t develop this problem yourself’* to the ‘perceived dangerous-not-weak’ factor did not alter the current findings for this factor. Reavley et al. [48] found ‘*you would not tell anyone if you had a problem like John’s’* to load moderate onto dangerous/unpredictable. In contrast our study found a moderately significant loading onto ‘perceived weak not sick’ but no loading onto the personal factor. While there is some differentiation between some of the variables associated with the personal and perceived stigma items, the social distance items appear to consistently load relatively uniformly onto a single factor across studies [45, 46].

Based on the findings of others [45], and of the principal component analysis this study measured a social distance score, a total personal stigma and total perceived stigma score in addition to ‘personal weak-not-sick’, ‘personal dangerous/unpredictable’, ‘perceived weak-not-sick’ and ‘perceived dangerous/unpredictable’ scores. ‘Personal weak-not-sick’ included “*John could make himself better if he wanted*‘, ‘*John’s problem is a sign of personal weakness*’, ‘*John’s problem is not a real medical illn*ess’. ‘Personal dangerous/unpredictable’ included ‘*John is dangerous*’ and ‘*John’s problem makes him unpredictabl*e’. ‘Perceived weak-not-sick’ and ‘dangerous/unpredictable’ used the same items presented in relation to what the participant felt most other people believe. Social distance items loaded consistently as described above. The perceived stigma items ‘*you would not tell anyone if you had a problem like John’s’* loaded moderately onto ‘perceived weak not sick’ and ‘*it is best to avoid John so that you don’t develop this problem yourself*’ loaded ‘moderately onto perceived dangerous/unpredictable’. However given the very moderate loading, and to ensure consistency with other studies [27] these items were not included in the final analysis of factors. Table 1 describes the Principal Component Analysis.

**Table 1:** Principal component factor analysis

	Social distance	Perceived stigma (weak not sick)	Perceived dangerous/unpredictable	Personal weak not sick	Personal dangerous not sick	No factor
Would you be happy to:						
Go to Johns house	.852					
Go out with John on the weekend	.828					
Develop a close friendship with John	.819					
Invite John around to your house	.805					
Work on a project with John	.642					
..tell us about what you think MOST OTHER PEOPLE believe						
Johns problem is Not a real medical illness		.784				
Johns problem is A sign of personal weakness		.774				
John could make himself better if he wanted		.797				
You would not tell anyone if you had a problem like Johns		.522				.241
John is dangerous			.746			
John’s problem makes him unpredictable			.748			
It is best to avoid John so that you don’t develop this problem yourself		.464	.553			
Indicate how Strongly YOU PERSONALLY agree/disagree with the statement						
Johns problem is Not a real medical illness				.830		
Johns problem is A sign of personal weakness				.838		
John could make himself better if he wanted				.609		
You would not tell anyone if you had a problem like Johns					.302	.642
John’s problem makes him unpredictable					.802	
John is dangerous					.754	
It is best to avoid John so that you don’t develop this problem yourself						.807

Chi square analysis was conducted to test associations of categorical variables between the intervention and control group. Intention to treat analyses were performed. Multiple imputation was used to impute variables for participants who completed baseline questionnaires but did not complete T_2_ and/or T_3_ questionnaire. Missing values were computed using baseline values. Repeated measures ANOVA was employed to determine if time (T_1_, T_2_ and T_3_) and group (intervention and control) had a significant effect on the outcome variables [38]. Between and within subject analyses were conducted. Results are reported considering the interaction between time and group. Partial eta squared was used to determine effect size. Data were analysed using SPSS Version 22. Significance level was set at 5%.

## Results

A total of 200 nursing students registered for the trial and after initial drop out and removal of ineligible students (*n* = 19), 181 students were randomised into either the intervention (*n* = 92) or control (*n* = 89) group. The baseline questionnaire (T_1_) was completed by 140 participants (intervention *n* = 59; control *n* = 81). At follow up (T_3_), 22% of the sample was lost to attrition, providing a final sample of 109 (intervention *N* = 50; control *n* = 59). The final sample (T_3_) did not differ significantly according to age, gender*,* country of birth or domestic or international enrolment status to the participants at T_1_. Intervention and control group participants did not differ significantly on any demographics. There were no significant differences for demographics for participants lost to the study at T_2_ or T_3_ apart from gender. Males were proportionally more likely to be lost from the study at T_2_, however females were more likely to represent those lost at T_3_. Participant demographics are described in Table 2 and the trial schema in Fig. 1.

**Table 2:** Participant characteristics at baseline (*n* = 140)

Demographic characteristics	Intervention n (%)	Control n (%)	Total (%)	*P* value
Gender				0.434
Male	8 (13.6)	15 (18.5)	23 (16.4)	
Female	51 (86.4)	66 (81.5)	117 (83.6)	
Age				0.921
18–24	47 (79.6)	59 (72.8)	106 (75.7)	
25–30	6 (10.2)	10 (12.3)	16 (11.4)	
31–35	1 (1.7)	6 (7.4)	7 (5)	
36–40	2 (3.4)	1 (1.2)	3 (2.1)	
41+	3 (5.1)	5 (6)	8 (5.7)	
Student status				0.412
Domestic Student	34 (57.6)	41 (50.6)	75 (53.6)	
International Student	25 (42.4)	40 (49.4)	65 (46.4)	
Enrolment Status				0.400
Full time	54 (91.5)	77 (95.1)	131 (93.6)	
Part time	5 (8.5)	4 (4.9)	9 (6.4)	
Previous mental health training				0.177
Yes	3 (5.1)	1 (1.2)	4 (2.9)	
No	56 (94.9)	80 (98.8)	136 (97.1)	

### Knowledge

Knowledge scores were significantly higher for the intervention at T_2_ compared to the control group when T_1_, T_2_ and T_3_ were compared. Both between-subject (df = 1, *F* = 0.320, *p* < .001, η^2^_*p*_ = 0.03. and within-subject differences were significant (df = 2, *F* = 21.00, *p* < .001, η^2^_*p*_ = 0.132) when T_2_ and T_3_ knowledge scores were compared to T_1_ scores. The intervention group reported an increase in mean knowledge score from 11.76 (T_1_) to 14.69 (T_2_) with mean scores reducing very slightly at T_3_ (13.51) (Table 3).

**Table 3:** Changes in knowledge, confidence, stigmatising attitudes and social distance

	T_1 (*n* =140)_		T_2 (*n* = 119)_		T_3 (*n* = 109)_		
	Intervention	Control	Intervention	Control	Intervention	Control	*P* value (Group x Time)
	Mean						
Knowledge *N* = 140	12.02	12.21	14.93	12.66	13.62	12.72	0.001**
Confidence *N* = 140	3.14	3.27	4.24	3.25	4.38	3.72	0.001**
Mental health intentions *N* = 140	3.40	3.37	5.44	3.73	4.93	3.16	0.001**
Social Distance *N* = 140	3.67	3.92	3.81	3.85	4.07	3.88	0.008*
Personal stigma (total) *N* = 140	3.58	3.60	3.93	3.73	4.01	3.81	0.008*
Personal weak not sick *N* = 140	350	3.66	3.81	3.80	3.94	3.88	0.110
Personal dangerous/ unpredictable *N* = 140	3.17	2.99	3.63	3.18	3.68	3.32	0.016*
Perceived stigma (total) *N* = 140	2.54	2.51	2.49	2.56	2.31	2.53	0.163
Perceived weak not sick *N* = 140	2.35	2.35	2.38	2.59	2.14	2.53	0.065
Perceived dangerous/ Unpredictable *N* = 140	2.59	2.49	2.47	2.44	2.21	2.44	0.247

*P values represent differences between intervention and control groups across the three time periods (Group x Time).*

*p < 0.05, **p < 0.001

### Confidence helping John

Intervention group participants reported significantly higher confidence scores both within (df = 2, *F* = 24.809; *p* < .001, η^2^_*p*_ = 0.153) and between (df = 1, *F* = 23.551; *p* < .001, η^2^_*p*_ = 0.149) subjects compared to the control group when T_1_ scores were compared with T_2_ and T_3_ scores (Table 3). Confidence in helping scores continued to increase among intervention group participants between T_2_ and T_3._

### Mental health first aid intentions

Intervention group participants were significantly more likely to report appropriate mental health first aid intentions towards helping John compared to the control group at T_2_ and T_3_ both (within-subjects: df = 2, *F* = 14.058, *p* < 0.01, η^2^_*p*_ = 0.093; between-subjects: df = 1; F = 32.466, *p* < 0.01, η^2^_*p*_ = 0.192). The intervention group reported an increase in mean mental health intention scores from 3.40 (T_1_) to 5.44 (T_2_) with mean scores reducing very slightly at T_3_ (4.93) (Table 3).

### Stigmatising attitudes and social distance

There was a significant difference between groups and time s over the three time periods for personal stigma (df = 2, *F* = 4.929; *p* = 0. 008, η^2^_*p*_ = 0.035) with participants from the intervention group reporting more positive levels of overall personal stigma at each time period compared to T_1._ Personal stigma scores continued to increase between T_2_ and T_3_ (Table 2). ‘Personal dangerous/unpredictable’ stigma scores also significantly increased indicating lower levels of stigma for intervention group participants at T_2_ and T_3_ compared to T_1_ in comparison to the control group (within-subjects: df = 2, *F* = 4.184; *p* = 0.016, η^2^_*p*_ = 0.030; between-subjects: df = 1, *F* = 7.785; *p* = 0.006, η^2^_*p*_ = 0.054). However there were no significant differences between intervention and control groups when T_1_ was compared to T_2_ and T_3_ for ‘personal weak-not-sick’ stigma (within-subjects df = 2, *F* = 2.227, *p* = 0.110, η^2^_*p*_ = 0.003; between-subjects; df = 1, *F* = 0.040, *p* = 0.842, η^2^_*p*_ = 0.000). Similarly there was no significant differences between group and time for perceived stigma (within-subjects: df = 2, *F* = 1.825; *p* = 0.163, η^2^_*p*_ = 0.013; between-subjects: df = 1, F = 1.401; *p* = 0.239, η^2^_*p*_ = 0.010), ‘perceived weak-not-sick’ (within-subjects: df = 2, *F* = 2.7549; *p* = 0.0654, η^2^_*p*_ = 0.020; between-subjects: df = 1, *F* = 2.817, *p* = 0.096, η^2^_*p*_ = 0.020) or ‘perceived dangerous/unpredictable’ stigma scores (within-subjects: df = 2, *F* = 1.405; *p* = 0.247, η^2^_*p*_ = 0.010; between-subjects: df = 1, *F* = 0.022; *p* = 0.881, η^2^_*p*_ = 0.000).

There was a significant difference when groups and time were considered for social distance scores (df = 2, *F* = 4.916, *p* = 0.008, η^2^_*p*_ = 0.035), While mean scores for control group participants remained similar, intervention group mean scores increased at each time period indicating better social distance at T_3_ compared to T_1_ (Table 3).

### Recognition of depression

The intervention group was more likely to positively identify John’s condition as depression compared to the control group at all three time periods, however this difference was not statistically significant (*F* = 0.102, *p* = 0.750) (Table 4).

**Table 4:** Correct identification of John’s condition (recognition of depression)

	T_1_ (*N* = 140)	T_2_ (*N* = 119)	T_3_ (*N* = 109)
Intervention	54 (91.5%)	49 (94.2%)	48 (94.1%)
Control	69 (85.2%)	60 (88.2%)	51 (87.9%)

## Discussion

This randomised controlled trial found face-to-face MHFA training for nursing students to be effective in changing knowledge, confidence in helping, mental health first aid intentions, social distance and overall personal stigma and personal dangerous/unpredictable stigma scores. MHFA facilitates knowledge and skills to enhance mental health literacy and enable participants to apply a mental health first aid action plan. Evaluations of MHFA have found positive changes to knowledge and awareness of mental health problems [27, 29, 49] (29) and improving confidence in help seeking and reducing stigmatising attitudes [36, 49, 50].

Similar to other studies this study found a significant increase in knowledge scores at post intervention (T_2_) which only slightly decreased at follow up (T_3_). A recent Australian study of online and face-to-face MHFA courses for university nursing and medical students found an increase in mental health knowledge, however did not use a control group [27]. A RCT measuring the impact of MHFA among teachers [29] found a significant increase in the intervention group compared to the control group, although, similar to the current study, there was some attenuation in knowledge at follow up compared to post intervention. The likelihood for attenuation in knowledge over time and the importance of the combination of knowledge, attitude and skill development to effect change [51] highlights the need for regular professional development for knowledge, attitude and skill maintenance. Although significant, knowledge scores remained relatively low, however they are comparative to those of other Australian studies [27, 29]. To date studies of MHFA have not measured retention of knowledge, recognition of depression, confidence, stigmatising attitudes or social distance over the longer term.

Recognition of depression was high at baseline for both intervention and control group participants. Although no intervention effect was found, recognition did improve among intervention group participants. At baseline (T_1_) and follow up (T_3_) 91.5% and 94.1% of intervention group students respectively correctly identified John’s condition as depression. The proportion of participants reporting correct recognition did not differ at T_2_ and T_3_ demonstrating good retention. Intervention effects may have been influenced by high baseline recognition among intervention and control group students which may be associated with participants studying nursing. Another study of nursing students found recognition of depression to be high at pre-course (face-to-face, 92.4%; online, 90.1%) and the study found no significant effects as a result of the course [27]. In comparison, baseline levels of recognition have been found to be lower among other groups completing MHFA training such as a broader university community (74% of students and 77% of staff) [6] and an Australian rural community (68% of intervention group participants and 74% of control group participants) [31]. A sample of young Australians aged 18–25 years who were not participating in a MHFA course found 58.6% were able to correctly identify depression using the vignette [52].

The MHFA training was effective in increasing the confidence of intervention group participants to help John. Confidence in supporting and help seeking is a key component of the MHFA course [36, 37, 50]. Similar to this study, other nursing and medical students have also reported increased confidence when presented with the ‘John’ vignette [27]. Teachers completing the course were also significantly more likely to report feeling confident in helping students and colleagues [29].

This study reported a significant improvement in mental health first aid intentions for intervention compared to control group participants. Similar to this study, Bond and colleagues reported nursing students’ mean mental health first aid intention scores to improve significantly for those completing online and face-to-face MHFA training [27].

Consistent with other research [26, 27] this intervention found a significant positive influence on social distance scores with respondents reporting lower levels of desire for social distance at post-test and follow up. There was also a significant positive intervention effect over time for personal stigma and ‘personal dangerous/unpredictable’ stigma. Scores for both groups demonstrated low levels of stigma at baseline which may be a characteristic of the nursing student population. Mental health professionals have been found to have lower levels of ‘personal weak-not-sick’ and ‘dangerous/unpredictable’ stigma compared to general community members [48]. Bond et al. [27] found a significant improvement in ‘personal weak-not-sick’ and ‘personal dangerous/unpredictable’ scores for both face-to-face and online nursing students at post intervention and at follow up. Another Australian study comparing data from 2003/04 to 2011 found increases in population based mental health literacy and personal contact with a person suffering depression while the desire for social distance decreased [53]. While greater awareness of depression appears to have impacted positively on social distance, beliefs that a person suffering depression was likely to be dangerous and unpredictable increased. This may be associated with awareness generated from campaigns and media stories [53]. Young people’s stigmatising attitudes have been found to influence first aid actions with those who reported higher levels of ‘dangerous/unpredictable’ stigma based on the vignette being more likely to suggest they would make a doctor’s appointment for John. In comparison, higher levels of social distance and ‘weak-not-sick’ dimensions predicted less helpful first aid responses [54]. These community-based findings highlight the importance of evidence based courses such as MHFA training to increase mental health literacy and improve help seeking actions [49].

While the Australian study discussed above demonstrated increases in mental health literacy and some levels of stigma reduced at a population level [53], a European study found despite increases in mental health literacy over an 11 year period, the desire for social distance from people with schizophrenia and depression remained unchanged [55]. It is recognised that stigma is a complex and multilayered phenomenon [54, 56] which may explain the limited change in some stigma scores in this study.

Consistent with the findings of another MHFA evaluation [27] this study found no significant intervention effect over time for the total perceived stigma of perceived ‘weak-not-sick’ or ‘dangerous/unpredictable’ factors. A RCT evaluating the impact of MHFA on teachers analysed each item for perceived stigma separately and found no significant intervention effect for any of the individual items apart from ‘other people would not tell anyone’ [29]. It is recognised that personally held stigmatising items have a distinct dimensions compared to perceived stigma [48] and the aim of MHFA is to increase health literacy and to change participant’s attitudes, as opposed to changing how participants perceive others to think [27].

A cross-sectional study of health professionals found psychologists to be less likely to hold personal stigmatising attitudes or a desire for social distance when compared to general practitioners. However, the study found although stigmatising attitudes were lower among health professionals compared to the general community, the difference between social distance scores were not significant [48].

### Limitations

This study had an overrepresentation of female participants (83.6%) when compared to the overall Australian university population estimate (57%) [57]. However this overrepresentation is reflective of the nursing workforce (91.2%) [58] and is consistent with the study by Bond et al. of nursing students (91% female) [27]. While this study included a higher proportion of international students (46.4%) than the national university average (approximately 22%) [59] these findings are consistent with the enrolment profile in the School of Nursing, Midwifery and Paramedicine at this university (2015: 43% international enrolments) [60].

The study population is not representative of the general population. Nursing students are likely to have better knowledge regarding mental health problems compared to the general population. To reduce this bias first year students were selected to participate in this study. However given the study population social desirability may have threatened internal validity of the study [38]. Blinding of participants was not possible due to logistics of organising the course hence participants were aware they had been allocated to intervention or control group. This may have also impacted social desirability of responses [38]. Study contamination may have occurred as all participants were nursing students enrolled at the same university. This is recognised as a limitation of this study [41].

Drop out once allocated to a condition was higher among participants in the intervention group compared to the control group. This may have been associated with the need to complete the course during semester. Control group participants were wait listed hence could select from a range of MHFA courses after the study period.

Demographic data were not collected from randomised participants who did not complete the baseline questionnaire hence Intention to Treat analysis was conducted using the 140 baseline participants. This is a limitation of this study.

Although significant changes were found for intervention compared to control group participants for mental health literacy, confidence, mental health intentions, social distance and some stigma these changes were not large. Additional supplements to the training over a longer period of time may be necessary for greater changes.

While other studies have employed follow up of 6 months [27], follow up in this study was limited to two months after the intervention due to academic timetabling constraints limiting access to participants over a longer term. After randomisation, the intervention group recorded some dropout due to clinical rotations as some students were unable to attend both days of the MHFA course, however there was little difference in demographics among those who were lost to the study at T_2_ and T_3._ This could be overcome by embedding MHFA into a specific unit or offering the course during semester breaks.

## Conclusion

MHFA can positively impact on mental health knowledge, confidence in helping, mental health first aid intentions, social distance and some aspects of personal stigma among nursing students. This course is especially relevant to nursing and other allied health students who are likely to benefit personally and professionally from the training. Providing opportunities for university students from a range of disciplines to complete MHFA courses as part of their university experience should be explored. This study did not have the capacity to measure longer term impact of the course. Following students into their second or third year of study and into the workplace to determine implementation of skills developed as part of the course should be explored. Additionally, the longer term impact of the training on participants applying skills developed should be explored. Enhanced mental health literacy, including help-seeking and lower levels of stigmatising attitudes will be beneficial to all young adults personally and in their chosen profession. University provides a structured setting to target young adults at the time when they are most likely to experience a mental health problem, or to have peers experiencing problems.
